# Effect of γ-tocotrienol in counteracting oxidative stress and joint damage in collagen-induced arthritis in rats

**DOI:** 10.3892/etm.2014.1592

**Published:** 2014-02-28

**Authors:** AMMU RADHAKRISHNAN, DULANTHI TUDAWE, SRIKUMAR CHAKRAVARTHI, GAN SENG CHIEW, NAGARAJA HALEAGRAHARA

**Affiliations:** 1 Faculty of Medicine and Health Sciences, International Medical University, Kuala Lumpur 57000, Malaysia; 2 Faculty of Medicine and Health Sciences, Universiti Tunku Abdul Rahman, Kuala Lumpur 57000, Malaysia; 3 Discipline of Physiology and Pharmacology, School of Veterinary and Biomedical Sciences, James Cook University, Townsville, Queensland 4811, Australia

**Keywords:** rheumatoid arthritis, oxidative stress, tocotrienols, tumor necrosis factor

## Abstract

Tocotrienols exhibit a significant anti-inflammatory and antioxidant effect in numerous human diseases. However, the anti-inflammatory and antioxidant effects of tocotrienols in arthritic conditions are not well documented. Therefore, the effect of γ-tocotrienol supplementation against oxidative stress and joint pathology in collagen-induced arthritis in rats was investigated in the present study. Adult female Dark Agouti rats were randomly divided into groups: Control, γ-tocotrienol alone, arthritis alone and arthritis with γ-tocotrienol. Arthritis was induced using 4 mg/kg body weight collagen in complete Freund’s adjuvant. The rats were treated orally with 5 mg/kg body weight of γ-tocotrienol between day 21 and day 45. After 45 days, serum C-reactive protein (CRP), tumor necrosis factor (TNF)-α, superoxide dismutase (SOD) and total glutathione (GSH) assays were conducted. γ-tocotrienol significantly reduced the arthritis-induced changes in body weight, CRP, TNF-α, SOD and the total GSH levels. There was a significant reduction in the arthritis-induced histopathological changes in the γ-tocotrienol treatment group. The data indicated that administration of γ-tocotrienol resulted in a significant antioxidant and anti-inflammatory effect on collagen-induced arthritis; therefore, γ-tocotrienol may have therapeutic potential as a long-term anti-arthritic agent in rheumatoid arthritis therapy.

## Introduction

Rheumatoid arthritis (RA) is a chronic inflammatory autoimmune disease that is associated with progressive disability and systemic complications (1,2). RA is able to initiate in any joint, however, it commonly begins in the smaller joints of the fingers, hands and wrists. Other joints that are commonly affected include the hips, knees, ankles, feet, neck, shoulders and elbows. In addition to joint pain and inflammation, individuals suffering from RA may experience fatigue, occasional fevers and a general sense of ill health (2–4). The cellular and molecular pathology of RA involves chronic inflammation of the synovium as well as synovial proliferation and infiltration by macrophages, memory T cells and plasma cells. Furthermore, marked hyperplasia of the synovium and progressive cartilage destruction occurs, which are mediated by cytokine-induced degradative enzymes (4,5). Regardless of recent improvements in the treatment options for RA, the pathology underlying inflammatory arthritis and its causative factors have not been well described.

Selection of the appropriate medication for RA is difficult due to the range of factors that contribute to the development of the disease. The drugs used for RA treatment include disease-modifying anti-rheumatic drugs, such as methotrexate, sulfasalazine, hydroxychloroquine sulfate and azathioprine (5–7). These may also be described as slow-acting anti-rheumatic drugs; they suppress inflammation and may impede the development of joint erosion. The mechanism by which the drugs act in patients with arthritis is not currently well understood; the majority of these drugs do not effect the progression of the disease; however, they may relieve the arthritic symptoms. The administration of these drugs is often limited due to an increased risk of cardiovascular events and upper gastrointestinal complications, such as gastric ulcers (4–6). The long-term effects of the anti-inflammatory therapeutic agents on the joint require further investigation; there is significant interest in supplements, nutraceuticals and novel therapeutic agents, which have the potential to reduce arthritic symptoms and impede the progression of the disease.

Vitamin E includes a family of lipophilic micronutrients consisting of four forms of tocopherols and tocotrienols (α, β, γ and δ), which consist of a chromanol ring and a side chain. Tocopherols and tocotrienols are found in various components of the human diet (8–9); tocopherols are primarily present in nuts and vegetable oils, while tocotrienols are minor plant constituents particularly abundant in rice bran, cereal grains and palm oil. Tocopherols have a saturated phytyl tail, whereas tocotrienols have an unsaturated phytyl tail. The individual isoforms of tocopherols and tocotrienols differ in the number and position of the methyl groups attached to the aromatic ring (10). The specific forms of vitamin E exhibit different biopotency; in the vitamin E group, α-tocopherol demonstrates the highest biological activity (11–12). Tocopherols have previously been investigated for their antioxidative, anti-inflammatory, anticancer and antineurodegenerative effects; however, investigations concerning the antioxidant and anti-inflammatory effects of tocotrienols are limited. γ-tocotrienols have recently become a point of interest due to improved therapeutic potential when compared with tocopherols. This specific isomer has been identified as exhibiting significant physiological activity within cell line and animal studies and γ-tocotrienol possesses antioxidant, anti-inflammatory, cardioprotective and neuroprotective properties (13,14). Previous studies have identified that the γ-tocotrienols and tocotrienol-rich fractions exhibit significant anti-inflammatory properties (13,15,16); however, to the best of our knowledge, no studies exist concerning the antioxidant and anti-inflammatory effects of γ-tocotrienols in arthritis. The present study investigated the anti-inflammatory and antioxidant properties of γ-tocotrienols against collagen-induced arthritis in Dark Agouti rats.

## Materials and methods

### Chemicals

Complete Freund’s adjuvant (CFA), type II collagen and γ-tocotrienol were purchased from Sigma-Aldrich (St. Louis, MO, USA). ELISA kits, obtained for the determination of superoxide dismutase (SOD) and total glutathione (GSH), were purchased from the Cayman Chemical Company (Ann Arbor, MI, USA). A C-reactive protein (CRP) assay kit was purchased from AssayPro (St. Charles, MI, USA) and a tumor necrosis factor (TNF)-α assay kit was obtained from eBioscience (San Diego, CA, USA). The remaining chemicals and reagents were obtained from Sigma-Aldrich (St. Louis, MO, USA).

### Animals

Female dark Agouti rats (age,10 weeks; weight, 120–140 g) were obtained from the Institute of Medical Research (Kuala Lumpur, Malaysia). The rats were housed in individual ventilation cages with food and water provided *ad libitum*. The experimental procedures were conducted according to internationally approved ethical guidelines for the care of laboratory animals and study gained approval from the International Medical University, Kuala Lumpur research and ethics committee.

The animals were randomly assigned into the following groups: i) Control; ii) arthritis; iii) γ-tocotrienol alone; and iv) arthritis with γ-tocotrienol.

### Collagen-induced arthritis

Following an acclimatization period, the rats were injected with collagen that was emulsified in CFA, as reported in previous investigations (17,18). Briefly, 5 mg collagen was dissolved in 2.5 ml cold, 0.1 M acetic acid. This mixture was emulsified with 2.5 ml CFA and the solution was mixed using a glass homogenizer (Fisher Scientific, Kuala Lumpur, Malaysia) for ~15 min. The procedure for preparing this solution was conducted on ice to ensure the proteins in the emulsion were not denatured. Prior to receiving the injection of the collagen-CFA mixture, the rats were anesthetized with diethyl ether (Sigma-Aldrich, St Louis, MO, USA) and 4 mg/kg body weight collagen-CFA emulsion was injected intradermally into the four paws of the rat as well as in to the base of its tail.

### γ-tocotrienol treatment

The rats in the γ-tocotrienol group were fed orally with 5 mg/kg of γ-tocotrienol from day 21 of the experiment and this treatment continued, by daily gavage, until day 45. The rats were fed normally and had access to water *ad libitum*.

### Evaluation of arthritis

The severity of arthritis was assessed via measurement of paw thickness, the changes were recorded from eight joints using a digital vernier caliper (TESA, Ludwigsburg, Germany). The thickness of two joints was measured in each of the four paws and the changes were recorded on alternating days.

### CRP assay

The CRP level in the plasma of the experimental animals was quantified using a rat CRP ELISA kit, in accordance with the manufacturer’s instructions. Briefly, the lyophilised biotinylated rat CRP was dissolved in 4 ml of enzyme immunoassay diluent solution and 25 μl of the diluted samples were added to the respective wells in duplicates. Subsequently, 25 μl diluted biotinylated rat CRP was added to each of the wells. The plate was incubated for 20°C 2 h; 50 μl diluted streptavidin peroxidase conjugate was added to each of the wells in addition to 50 μl chromogen substrate, which was added to each well to enable color development. The stop solution was added to each well and the absorbance was read at 450 nm using a microplate reader (TECAN, Männedorf, Switzerland); the CRP concentration of each sample was calculated based on the standard curve obtained.

### TNF-α assay

The concentration of TNF-α in the plasma was quantified using a rat TNF-α ELISA kit. Briefly, the plate was coated with the appropriately diluted capture antibody (pretitrated, purified antibody) one night prior to conducting the assay. On the following day, 100 μl standard solution and the sample was added to the respective wells in duplicate. Following this, 100 μl diluted detection antibody solution was added to the wells and the plate was incubated for 1 h. Diluted avidin-horseradish peroxidase (100 μl) was added to the wells and incubated for 30 min in the dark followed by the addition of 100 μl substrate solution. Stop solution, 2N (H_2_SO_4_; 50 μl), was added to the wells and the absorbance was read at 450 nm using a microplate reader. The standard curve was used to calculate the TNF-α levels in each sample.

### SOD assay

The SOD concentration was quantified using a SOD assay kit, in accordance with the manufacturer’s instructions. Briefly, the assay buffer, sample buffer, radical detector, xanthine oxidase (XO), plasma samples and standard solutions were prepared in accordance with the manufacturer’s instructions. Subsequently, 200 μl diluted radical detector was added to the wells and 10 μl standard solution was added to the relevant wells in duplicate. Diluted plasma samples (10 μl) were added to the relevant sample wells and the reaction was initiated by the addition of 20 μl XO into each well. The absorbance was read at 450 nm using a microplate reader. The standard curve was used to calculate the SOD level of each sample.

### GSH assay

The total GSH activity in the plasma of the experimental rats was quantified using a total GSH assay kit. Briefly, sample deproteination was conducted and 50 μl standard solution and 50 μl sample was added to the respective wells. Following this, 150 μl of the assay cocktail (mixture of N-morpholino ethanesulphonic acid buffer, NADP^+^ and glucose 6-phosphate, glutathione reductase and glucose 6-phosphate dehydrogenase mixture and 5,5′dithio-bis-2-nitrobenzoic acid) was added to each well. The absorbance was measured at time intervals of 5 min for a total of 30 min, using a microplate reader with a 450-nm filter. The absorbance value of the standard solution was subtracted from the values obtained from the standard and the sample solution. A graph was plotted with the corrected absorbance values of each of the standard solutions as a function of the concentration of total GSH, thus the total GSH value for each sample was calculated.

### Histopathological analysis

Following the sacrifice of the rats with overdose of anaesthesia (pentobarbital sodium), their joints were harvested, the flesh was removed from the bone and the joint samples were stored in 10% formalin solution for three weeks in specimen bottles. Blocks were prepared following the decalcification of the joints for 48 hours, the blocks were sectioned at 3–4 μm thickness and slides were prepared and stained with hematoxylin and eosin (H&E). The joints were evaluated and analyzed according to the grading system adopted in a previous study (19).

### Statistical analysis

Values were expressed as the mean ± standard error of the mean. The differences were analyzed for significance using one-way analysis of variance with Bonferroni post hoc multiple comparisons, which were used to assess the differences observed between the independent groups. P<0.05 was considered to indicate a statistically significant difference.

## Results

### Body weight

**Figure 1:**
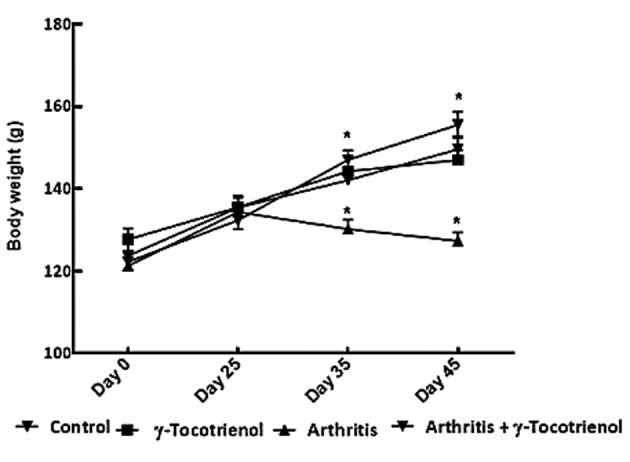
Body weight changes in the arthritis group and following γ-tocotrienol treatment. Data are presented as the mean ± standard error of six rats per group. ^*^P<0.05 vs. the control group.

There was a significant increase in body weight in the normal control group and in the group with γ-tocotrienol alone (P<0.05). The arthritis alone group exhibited a significant decrease in body weight throughout the duration of the experiment (P<0.05). On day 35 and 45, the arthritis group exhibited a significant decrease in body weight compared with the other groups (P<0.05; Fig. 1).

### Paw thickness

**Figure 2:**
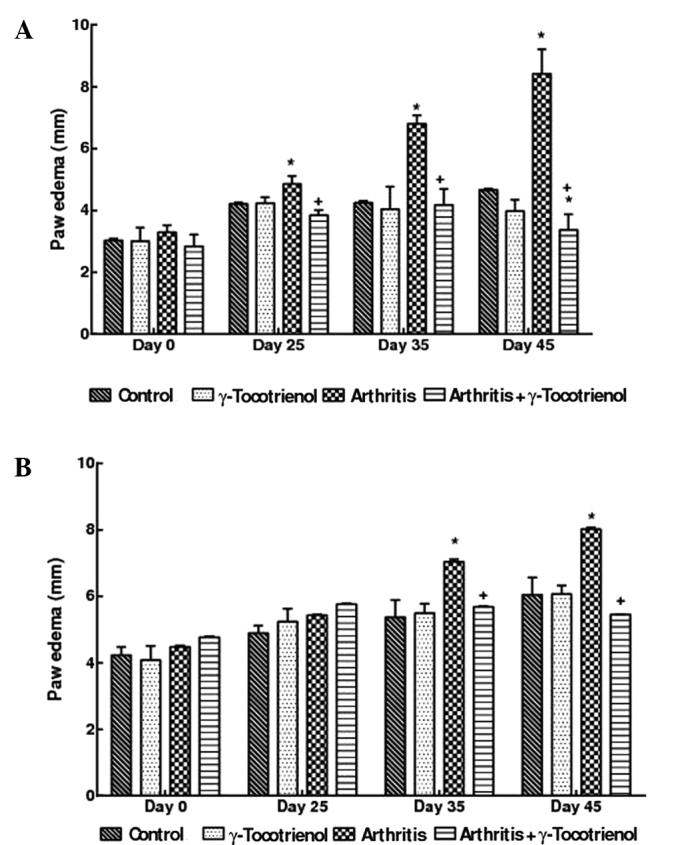
Effect of γ-tocotrienol on paw edema in the arthritis group. (A) Left paw and (B) Right paw. Data are presented as the mean ± standard error of six rats per group. ^*^P<0.05 vs. the control group and ^+^P<0.05 vs. the arthritis group.

**Figure 3:**
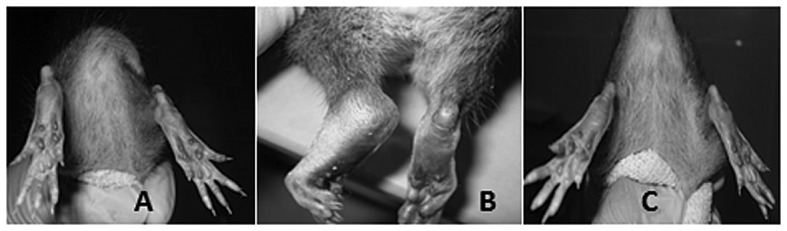
Effect of γ-tocotrienol on hind paw edema. (A) Control rats without arthritis without any paw edema; (B) Arthritis rats showing severe paw edema; (C) Arthritis rats treated with γ-tocotrienol exhibiting a significant reduction in the paw edema.

The arthritis alone group showed significant, macroscopic signs of severe arthritis such as swelling, redness, deformity and ankylosis in the hind paw and ankle joints; however, these symptoms were less pronounced in the forelimbs. There was a significant decrease in the hind paw thickness and edema of the g-tocotrienol treated arthritis rats (P<0.05) compared to arthritis alone rats. At the end of the experimental period, the γ-tocotrienol treated arthritis rats exhibited a hind paw thickness that was analogous to that of the normal control rats. No significant changes in paw thickness were observed in the γ-tocotrienol alone group and compared with the γ-tocotrienol alone group, the rats with arthritis and γ-tocotrienol (treatment from day 1) exhibited a significant reduction in paw thickness (P<0.05; Figs. 2A,B and 3).

### Plasma levels of CRP, TNF-α, SOD and GSH

**Figure 4:**
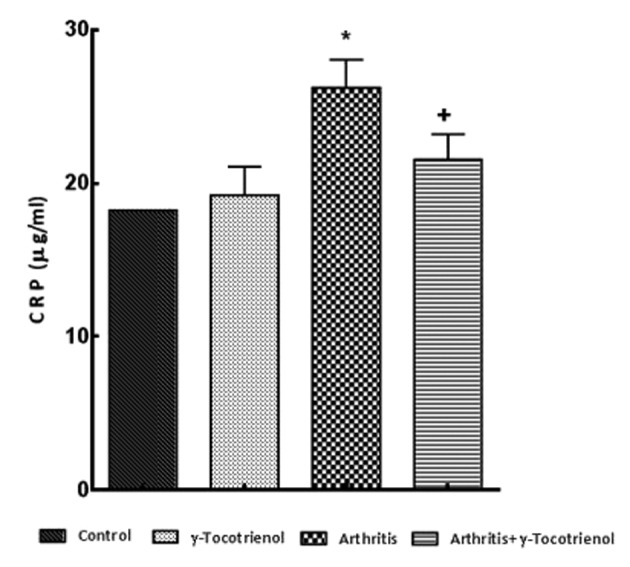
Effect of γ-tocotrienol on C-reactive protein (CRP) concentration. Data are presented as the mean ± standard error of six rats per group. ^*^P<0.05 vs. the control group and ^+^P<0.05 vs. the arthritis group.

**Figure 5:**
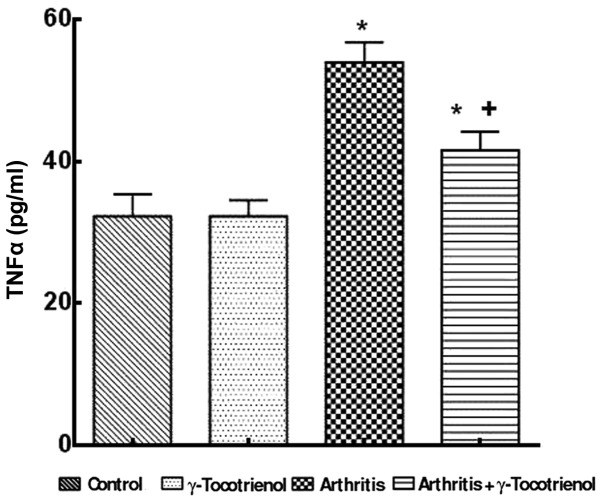
Effect of γ-tocotrienol on tumor necrosis factor-α (TNF-α) level. Data are presented as the mean ± standard error of six rats per group. ^*^P<0.05 vs. the control group and ^+^P<0.05 vs. the arthritis group.

**Figure 6:**
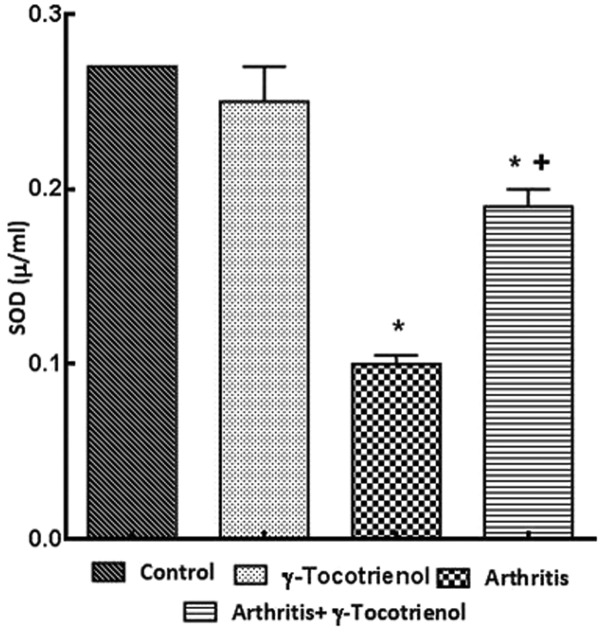
Effect of γ-tocotrienol on serum superoxide dismutase (SOD). Data are presented as the mean ± standard error of six rats per group. ^*^P<0.05 vs. the control group and ^+^P<0.05 vs. the arthritis group.

**Figure 7:**
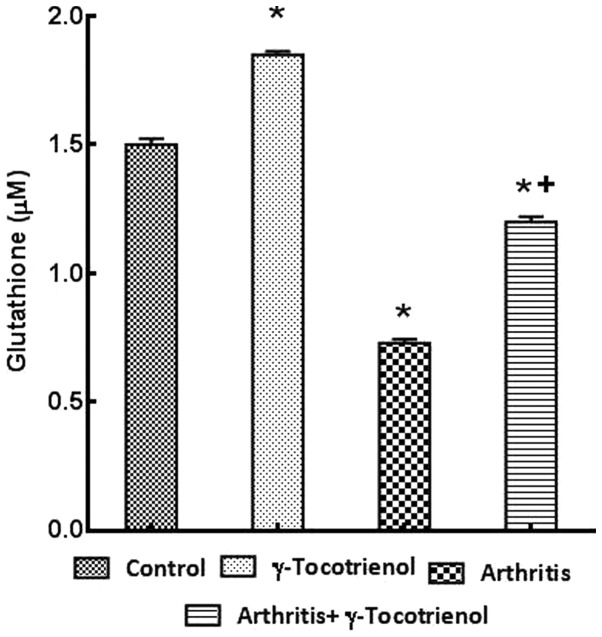
Effect of γ-tocotrienol on serum glutathione levels. Data are presented as the mean ± standard error of six rats per group. ^*^P<0.05 vs. the control group and ^+^P<0.05 vs. the arthritis group.

ELISAs were performed to quantify the CRP levels in the plasma. There was a significantly elevated CRP concentration observed in the untreated arthritis group and the arthritis group treated with γ-tocotrienol, when compared with the control rats (P<0.05). However, the CRP level was significantly decreased in the γ-tocotrienol group when compared with the CRP levels of the untreated arthritis group (P<0.05; Fig. 4). The untreated arthritis group showed a significantly higher concentration of TNF-α compared with the γ-tocotrienol-treated group (P<0.05). Treatment with γ-tocotrienol to arthritis rats resulted in a significant reduction in TNF-α when compared with the untreated arthritis group (P<0.05; Fig. 5). There was a significant decrease in the SOD in the arthritis group and the γ-tocotrienol-treated group indicated high levels of SOD concentration when compared with the arthritis only group (Fig. 6). The γ-tocotrienol-treated arthritis group exhibited significantly elevated levels of total GSH when compared with the arthritis group (P<0.05; Fig. 7).

### Histopathological analysis

**Figure 8:**
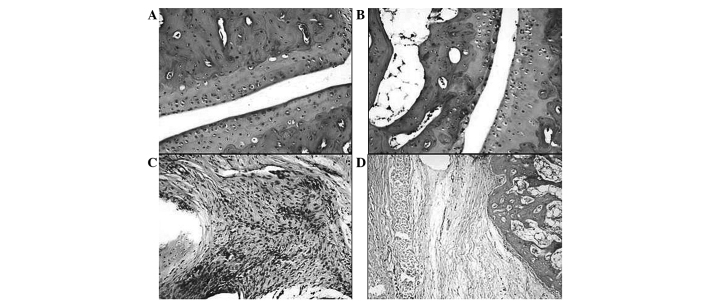
Histopathological analysis of joint morphology (magnification, ×200). (A) Control group exhibiting a normal joint. (B) γ-tocotrienol treatment group, exhibiting normal joint morphology. (C) Arthritis group exhibiting synovial fibrosis, congestion and hyperplasia. (D) Arthritis with γ-tocotrienol treatment group exhibiting mild synovial hyperplasia.

To evaluate the treatment of γ-tocotrienol against collagen-induced arthritis, histopathological analysis was conducted using an adapted method from a previous study (19). The arthritis only group showed a severity of grade three, while the γ-tocotrienol-treated arthritis group exhibited the characteristics of grade two severity. The significance between these groups, in terms of pathological conditions based on their grading, was calculated against the untreated arthritis group. The γ-tocotrienol-treated arthritis group exhibited a significant reversal in the histopathological changes compared with the untreated group (Fig. 8).

Synovial hyperplasia was observed in the untreated arthritis group; the rats appeared to develop extensive edema resulting in a narrowing of the joint space. There was inflammation, to the extent of forming panni, observed in numerous locations within the rats in the untreated arthritis group. The panni were composed of a granulomatous accumulation of chronic inflammatory cells, such as lymphocytes, plasma cells, macrophages and multinucleated giant cells. In the arthritis treated with γ-tocotrienol group, it was observed that synovial hyperplasia was moderately present in addition to inflammation and vascular dilation. The inflammation was moderate and was observed as scattered clusters of chronic inflammatory cells, with few focal attempts at granuloma formation. In the control group the fore- and hind limbs were identified as exhibiting a normal joint orientation. There was no evidence of edema, cellular infiltration, joint narrowing, synovial hyperplasia, fibrosis or erosion. In the untreated arthritis rats, the joints exhibited extensive edema with narrowing of the joint spaces and the surface of the joint margins exhibited degenerative changes. In the arthritis group treated with γ-tocotrienol there was narrowing of the joint space, however, it was to a lesser extent than that in the untreated arthritis group (Fig. 8).

## Discussion

The present study demonstrated that γ-tocotrienols are an effective inhibitor of arthritis-induced oxidative stress and TNF-α secretion. To the best of our knowledge, this is the first study to identify the anti-arthritic effect of γ-tocotrienols, against collagen-induced arthritis, in Dark Agouti rats. Female Dark Agouti rats, aged 6–10 weeks, were injected with type II collagen emulsified with CFA, which induced an immunological hypersensitivity reaction to the collagen within the rats, leading to the development of chronic inflammatory arthritis. The arthritis developed within 2–3 weeks of primary immunization and exhibited characteristic arthritic pathology comparable to that of human RA (20,21). A significant decrease in the body weight of the rats was observed in the arthritis group compared with the other groups, which confirmed the observations in previous studies where collagen-induced arthritis significantly decreased body weight, and where the body weight was reduced following three weeks of immunization (22,23). Body weight loss is the hallmark symptom of inflammatory arthritis, where a gradual decrease in weight gain is observed as the disease progresses (24). The γ-tocotrienol-treated arthritis group experienced a significant recovery of body weight following the second immunization. Therefore the γ-tocotrienol supplement to arthritis rats may have decreased the production of reactive oxygen species within the tissues and inhibited the metabolic rate of arthritic rats; γ-tocotrienol was able to impede the metabolism of the body, thus favoring fat accumulation (25).

CRP is an inflammatory marker, which is a member of the group of acute phase proteins and the level of CRP increases in response to inflammation (26,27). The CRP assay is used as an optimal laboratory test for the observation of inflammation resulting from RA and other inflammatory diseases. It is an effective indicator of tissue damage and the concentration of CRP in serum is associated with disease activity (27,28). In the present study, an increased CRP level was observed in the circulation of rats with arthritis and treatment with γ-tocotrienol significantly inhibited the arthritis-induced CRP changes observed. The higher level of CRP observed in the arthritis group confirmed the pathology of the joint and the CRP production may have increased as a result of the activated macrophages and fibroblasts within the synovium of the inflamed joints. The production of CRP is also controlled by inflammatory mediators within the joints including IL (interleukin)-1 and IL-6, thus the reversal of CRP levels following supplementation indicates a significant decrease in the activation of synovial macrophages and fibroblasts (29).

Various inflammatory mediators are released, which are responsible for pain in addition to swelling in the joints observed in cases of severe arthritis. The most common inflammatory mediators are IL-1β and TNF-α (30,31). A series of inflammatory changes develop following the administration of collagen in arthritic rats; joint swelling, infiltration of inflammatory cells, bone destruction and cartilage erosion were the significant arthritic changes that were observed in the present study. In inflammatory arthritis, CD4^+^ T helper cells are activated in the joints that stimulate the production of cytokines and other inflammatory mediators. TNF-α is produced by macrophages and the synovial lining, and is present at higher concentration in individuals suffering from arthritis; TNF-α modulates the secretion of proinflammatory cytokines (IL-1 and IL-6) within the synovial joints (31–33). TNF-α acts synergistically with IL-1β in the production of matrix metalloproteinases, the expression of cell adhesion molecules and the secretion of prostaglandins and these changes result in the joint destruction that is associated with arthritis. The present study demonstrated that γ-tocotrienol supplementation in arthritic conditions attenuated the arthritis-induced elevation of the TNF-α level. Furthermore, activation of transcription factor, nuclear factor kappa-light-chain-enhancer of activated B cells (NF-κB) is considered to be key in TNF-α-induced inflammatory processes, including the upregulation of IL-6. In previous studies, γ-tocotrienol was shown to exhibit an inhibitory effect on the NF-κB activation pathway (33–35). In addition, Wu *et al* (2008) identified that the tocotrienol-rich fraction was capable of inhibiting proinflammatory cytokines in human monocyte cells (36). Non-steroidal anti-inflammatory drugs, glucocorticoids and other immunosuppressants, that are commonly used in the treatment of RA, inhibit the NF-κB pathway and the expression of different inflammatory-associated genes. At present, inhibitors of NF-κB are considered to be the optimum anti-inflammatory drug in the therapeutic treatment of arthritis (33,37). In the present study, γ-tocotrienol significantly inhibited the TNF-α level observed in the circulation of the rats, which may be a result of its suppressive effect on the activation of the NF-κB pathway within the joints. The findings provide support for the use of γ-tocotrienol as an anti-inflammatory candidate for the treatment of arthritis; moreover, to the best of our knowledge, there are no known side effects as a result of prolonged treatment.

Free radicals are significant in the induction of RA (38); activation of mono- and polymorphonuclear cells in the articular joints result in oxidative damage within the joints. Increased oxidative stress is indicated by decreased concentrations of SOD and total GSH; two significant antioxidant enzymes within the circulation. A case of chronic inflammatory arthritis reduces the antioxidant capacity of the body and leads to an imbalance in the oxidant-antioxidant system (39,40). The significant decline in the level of SOD and GSH in the present study indicated an increase in the accumulation of the reactive oxygen species within the synovium and that these antioxidant enzymes were depleted due to quenching of the free radicals (41). Tocotrienols possess a potent antioxidant property, thus treatment with γ-tocotrienol enabled an increase in SOD and total GSH levels in the blood, which aided with reducing oxidant-induced joint tissue damage. Furthermore, tocotrienols exhibit superior antioxidant and anti-lipid peroxidation effects when compared with tocopherols, therefore tocotrienols have gained interest. Previous studies identified that low doses of tocotrienols were exhibiting an improved antioxidant and free radical scavenging effect, when compared with α-tocopherols (42,43). γ-tocotrienol exhibits significant antioxidant activity due to an ability for greater distribution within the membrane bilayer (15,41,43). It exhibits an improved ability to trap free radicals as a result of the unsaturated double bonds within the chemical structure. The restoration of the two antioxidant enzyme levels with γ-tocotrienol supplementation may be attributed to the ability of γ-tocotrienol to elevate the mRNA expression of these enzymes.

The histopathology of collagen-induced arthritis in Dark Agouti rats indicated cartilage destruction and extensive pannus formation, bone resorption and synovitis. Histopathological and biomarker changes correlated with the changes observed in paw edema. The suppression of vascularity, congestion, pannus formation and joint space narrowing, as a result of treatment, indicated the anti-arthritis effect of γ-tocotrienol. The γ-tocotrienols may have suppressed the progression of arthritis by inhibiting the chronic inflammatory phase and decreasing the free radical accumulation within the joints, thus reducing the incidence of cartilage destruction (6).

In conclusion, the results of the present study indicated that γ-tocotrienol was capable of reducing the oxidative stress and inflammation that was observed in the collagen-induced arthritic rats. The γ-tocotrienol treatment increased the antioxidant enzyme levels and decreased the TNF-α levels observed in arthritic rats, which provided protection against arthritis-induced joint damage. Histopathology indicated that the administration of γ-tocotrienol protected the joints and prevented the destruction of cartilage, thus significantly improving the arthritic symptoms. Therefore, γ-tocotrienol may be an effective, long-term anti-arthritic agent for reducing the serious side effects of synthetic, anti-arthritis drugs.
